# Self‐resolving bullous pemphigoid induced by cemiplimab

**DOI:** 10.1111/dth.15466

**Published:** 2022-03-29

**Authors:** Mattia Fabio Molle, Niccolò Capurro, Astrid Herzum, Claudia Micalizzi, Emanuele Cozzani, Aurora Parodi

**Affiliations:** ^1^ Section of Dermatology, Department of Health and Science (DissaL) Polyclinic Hospital San Martino, IRCCS, Università di Genova Genoa Italy

Dear Editor,

Cemiplimab was the first approved immunotherapy for advanced or metastatic cutaneous squamous cell carcinoma (cSCC) in patients not candidate to radical surgery or radiotherapy.^1^ Cemiplimab, a fully human IgG4 monoclonal antibody, exerts its anti‐tumor activity blocking programmed cell death protein‐1 (PD‐1). It has good safety/tolerability profile, but similarly to other immune checkpoint inhibitors, cemiplimab was associated with immune‐related (IR) adverse events (AE).^1^ We report a rare case of self‐resolving bullous pemphigoid (BP) induced by cemiplimab in an elderly patient treated for locally advanced cSCC.

An 83‐year‐old Caucasian man presented for new onset of generalized pruritus and cutaneous blisters. He had been administered cemiplimab for 18 months as adjuvant immunotherapy for invasive SCC (350 mg i.v. every 3 weeks). Clinical examination showed multiple, diffuse, tense bullae on erythematous skin, sparing mucosae (Figure 1A). Peripheral blood tests evidenced hypereosinophilia (2400 cells/μl) and elevated anti‐BP180 IgG (>200 RU/ml) at enzyme‐linked immunosorbent assay test, while anti‐BP230 IgG tested negative (ELISA‐Euroimmun).

**FIGURE 1:**
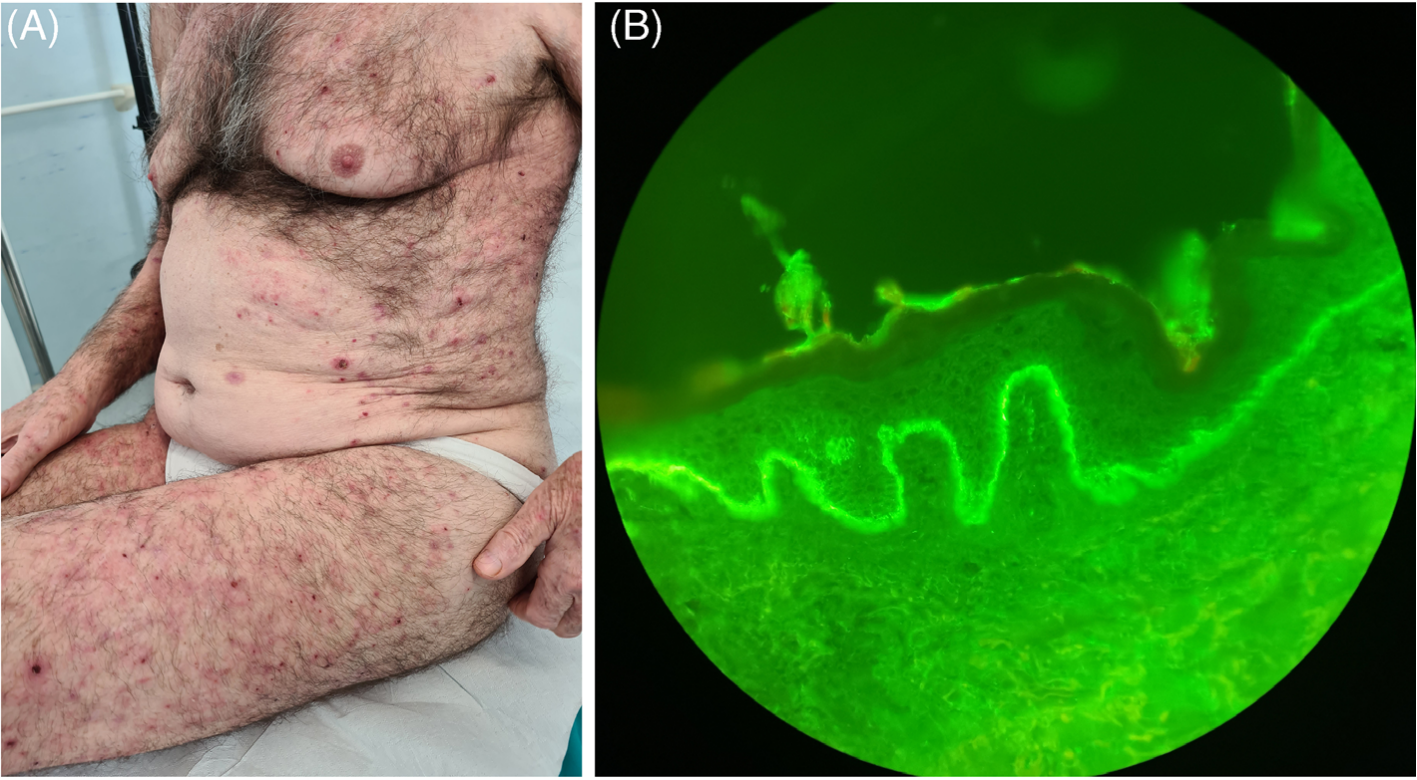
(A) Multiple erythematous plaques with blisters and erosions. (B) Direct immunofluorescence (DIF) showing C3 deposits at the dermoepidermal junction

Histopathology of perilesional skin evidenced subepidermal blistering, eosinophilic spongiosis and diffuse dermal eosinophils. Direct immunofluorescence showed linear IgG and C3 deposits at the dermal‐epidermal junction. The presence of weak perivascular deposits of IgA and IgM in the dermis has also been highlighted (Figure 1B). BP induced by PDL‐1 inhibitor was diagnosed and scored grade 2 in accordance with the Common Terminology Criteria for Adverse Events version 5. Cemiplimab was interrupted and application of clobetasol on severest lesions was prescribed. The patient interrupted topicals after only 2 days of clinical improvement.

After 2 weeks, no new bullae had formed; desquamation and hyperpigmentations were evidenced. Cemiplimab has demonstrated efficacy and acceptable safety profile in clinical trials, comparable to other anti PD‐1 drugs.^1^

The most common AE of cemiplimab, from its registration study EMPOWER‐CSCC 1, were rash, fatigue, diarrhea, pruritus, pneumonia and hepatitis. Also, though less frequently, IR‐AE were reported, as for other immune checkpoint inhibitors. Among IR‐AE, infusion‐related reactions, immune‐mediated pneumonia, hypothyroidism, and adverse skin reactions were described.^1^ Regarding the latter, two cases of cemiplimab‐induced BP have already been reported.^2^, ^3^

BP is the commonest autoimmune bullous disease, mostly affecting elderly population. BP is caused by autoantibodies directed against two haemidesmosomal proteins (BP 230 and BP180), producing tense subepithelial blisters involving skin and mucous membranes.^4^ BP can be induced by several classes of drugs, especially antibiotics, biologics, loop diuretics, anti‐diabetics, or anti‐inflammatory drugs.^4^, ^5^ The pathogenesis of drug‐induced BP is not yet fully established, however in genetically predisposed individuals the drug may induce an exaggerated immune reaction or an alteration of cutaneous basal zone antigenic properties, ultimately leading to autoimmunity.^4^

Inhibitors of PD‐1 and programmed death ligand‐1 (PD‐L1) increase inhibitory control of the immune system, particularly T‐cell suppression, eliciting enhanced anti‐tumor response. However, immune hyper‐activation can also produce IR‐AE, including BP. The two literature reported cases of cemiplimab‐induced BP, required steroid treatment for resolution. In the case reported by *Virgen* et al, the patient initially received systemic steroids, and discontinued cemiplimab. Nonetheless, blisters recurred: combined therapy with rituximab was necessary, finally obtaining clinical remission.^2^ In the case reported by Grünig et al., the patient was treated with systemic steroids and withdrawal of cemiplimab, with clinical remission.^3^

In our case, cemiplimab was discontinued and local steroid therapy was applied for 2 days. After 2 weeks, the clinical picture was clearly self‐improved: prior blisters resolved, no new blisters developed, erythema and itching diminished. The possible self‐resolving nature of drug‐induced BP has already been described as a consequence of the cessation of the immunostimulatory drug trigger.^6^

The mild perivascular dermal deposits of IgA and IgM, although constituting a finding of minor importance compared to the predominant deposition of IgG at the dermoepidermal junction, represent an unusual element which is difficult to interpret.

From a purely speculative point of view, we believe that the non‐specific immune activation of PD‐L1, which is known to cause numerous immune‐related adverse events, may induce vasculitis‐like changes of subclinical relevance.^4^

Our case not only consolidates the possibility that cemiplimab can induce BP, but also presents a hitherto unpublished self‐resolving variant. This information may help the clinician dealing with a similar situation to adopt a wait‐and‐see approach to avoid early treatment in elderly and frail patients, often with significant comorbidities.

## CONFLICT OF INTEREST

The authors declare no conflicts of interest.

## AUTHOR CONTRIBUTION

Study concept and design: Mattia Fabio Molle, Niccolò Capurro, Astrid Herzum, Claudia Micalizzi. Acquisition of data: Mattia Fabio Molle, Niccolò Capurro, Astrid Herzum, Claudia Micalizzi, Emanuele Cozzani, Aurora Parodi. Drafting of the manuscript: Mattia Fabio Molle, Niccolò Capurro, Astrid Herzum. Critical revision: Claudia Micalizzi, Emanuele Cozzani, Aurora Parodi.

## ETHICS STATEMENT

All the procedures adopted in the present study were in respect to the ethical standards in the World Medical Association Declaration of Helsinki. The subject gave his written informed consent to publish the present case (including publication of images).

## Data Availability

The data that support the findings of this study are available on request from the corresponding author. The data are not publicly available due to privacy or ethical restrictions.
